# Quantifying Global Foreign Affairs with a Multimodal Dataset of Diplomatic Websites

**DOI:** 10.1038/s41597-025-06334-5

**Published:** 2025-11-28

**Authors:** Nihat Muğurtay, Kaan Güray Şirin, Mehrdad Heshmat Najafabad, Ahmet Taha Kahya, Fazlı Göktuğ Yılmaz, Yasser Zouzou, Batuhan Bahçeci, Ayça Demir, Doğukan Tosun, Meltem Müftüler-Baç, Onur Varol

**Affiliations:** 1 Faculty of Arts and Social Sciences, Sabanci University, Istanbul, Türkiye; 2 Center of Excellence in Data Analytics, Sabanci University, Istanbul, Türkiye; 3 Faculty of Engineering and Natural Sciences, Sabanci University, Istanbul, Türkiye

**Keywords:** Politics, Social sciences

## Abstract

This research introduces a global dataset of diplomatic news and images compiled from the official webpages of ministries of foreign affairs and chief executive offices across 156 countries spanning over 20 years. The collection provides over 1.16 million news articles and 1.18 million associated images. Our research initially shows how web scraping and Natural Language Processing (NLP) tools enhance labor-saving, novel data acquisition and processing methods. First, we extracted named entities for people, countries, and organizations mentioned in diplomatic texts. Second, GlobalDiplomacyNET processes and analyzes images published on diplomatic webpages, capturing governments’ image-sharing practices. This textual and visual information together provides substantial information on countries’ news-sharing habits, geographical and multilateral attention, visual assertiveness, and gender representation. GlobalDiplomacyNET is the first of its kind, offering a global corpus of textual and visual data that support novel research directions particularly in international relations and political science.

## Background & Summary

Recent progress in novel computational techniques allows researchers to leverage large amounts of data available across disciplines and conduct studies at an unprecedented level by efficiently processing data and reducing manual effort^1^. These advanced computational techniques can be applied to in-press or online news, policy documents, social media posts, and leaders’ speeches^2–9^. In this context, International Relations (IR) and Political Science (PS) are becoming significant venues for researchers to apply computational tools^10^. We contribute to these efforts by introducing **GlobalDiplomacyNET** and providing an empirical understanding of the global inventory of diplomatic news. These textual data -acquired from governmental sources- will play a critical role in helping scholars extract useful information on cutting-edge topics in global affairs. Such news often includes statements, press briefs, meetings, summits, other political events, and diplomatic reactions revealing multiple dimensions of inter-state political behavior. Beyond textual data, countries also use diplomatic images as a strategic tool^11^, yet the quest for processing visual content in international relations remains underdeveloped despite its potential to reveal multiple image characteristics such as gender composition and other image attributes ^12^. **GlobalDiplomacyNET** captures information from 156 countries, spanning over 20 years and contains over 1.16 million reports and 1.18 million images. Focusing on the global corpora of diplomatic news (texts and images), we demonstrate how recent advancements in computational social sciences can enhance the study of global politics and foreign policy analysis.

Our research is structured around a three-step empirical agenda. First, we acquired all textual and visual content from countries’ chief executives and ministry of foreign affairs (MoFA) webpages, using automated web scraping methods. Second, GlobalDiplomacyNET uses Natural Language Processing (NLP) and computational image analysis to extract information from the data inventory for further analysis. These first two steps address the significant resource constraints traditionally associated with manual content analysis. Third, our research provides substantial information on countries’ political attention and focus, uncovering how countries pay special attention to certain geographies and organizations. Using named-entities for persons, we provide a snapshot of co-occurrence networks investigating which individuals co-occur in global diplomatic news. Our image analysis reveals significant disparities in how countries prioritize gender representation.

Our research contributes to previous work, especially those projects that process large amounts of texts and capture entities, mentions, and interactions from political news and texts. Global Database of Events, Language, and Tone (GDELT)^13^ has been processing a vast amount of news articles collected from different media outlets and online sources using machine learning algorithms. Scholars use similar data to capture top-trending political issues, including migration, political discourse^14^, and political violence forecasting^15^. Topic modeling also offers a significant venue for text-classification, and making it possible to extract political information^16,17^, particularly on parliamentary^18^ or executive speeches ^19^, tweets^20^, news outlets, and foreign policy documents^21^.

In recent years there has been an explosion in the number of texts obtained from diplomatic archives^22^. This trend has broadened the IR scholarship that analyzes large volumes of textual data. Historical diplomatic documents are processed and discussed in the realm of Foreign Policy Analysis (FPA)^23,24^ regarding relationship between countries’ diplomatic, political and economic behavior, with a limited number countries^25^. Acquiring and analyzing data from diplomatic archives has its own challenges^26,27^. Collecting and working with diplomatic data from open sources has traditionally entailed manual human labeling and coding. Computational techniques can overcome challenges of traditional archival research^28^. Our research demonstrates how NLP tools and LLMs can substantially reduce the human labor required to annotate political texts^29^. Further operationalization of our dataset can support these labor-intensive processes, including annotating and analyzing textual data to capture various types of information such as high-level diplomatic and economic interactions, text transcriptions, state recognition, and others^30–35^. In particular, the Correlates of War (Diplomatic Exchange)^31^, DIPLOMETRICS^36,37^, and DIPCON^38^ datasets are notable examples of diplomatic exchange and interaction datasets. GlobalDiplomacyNET goes beyond such diplomatic datasets. For instance, while a U.S. President or Secretary of State may not visit North Korea, and diplomatic exchanges between the two countries may be absent, North Korea can still be frequently mentioned in diplomatic texts. Asymmetry between diplomatic exchanges and mentions is also another research topic that can reveal multiple dimensions of *political attention*.

When it comes to computational applications in diplomatic relations, Diplomatic Pulse^39^ and GDELT emerge as promising examples of compiling global diplomatic information. However, GDELT’s event-centric data does not provide full-texts directly^40^. We find that 63,318 out of 1,163,905 urls matched entries in GDELT’s post-2015 data accessible through Google BigQuery. Country-level examinations further underline differences in coverage. For instance, among the 4,076 post-2015 URLs from the Turkish Ministry of Foreign Affairs (mfa.gov.tr), only 757 were present in GDELT; among the 9,728 URLs from the U.S. executive branch (whitehouse.gov), 3,938 were matched; and among the 10,910 URLs from the Russian Ministry of Foreign Affairs (mid.ru), 7,389 were found. These findings corroborate GDELT’s limited diplomacy-related news/data coverage, and demonstrate that coverage ratios vary considerably across countries. Diplomatic Pulse is closer to GlobalDiplomacyNET in terms of data coverage; however with GlobalDiplomacyNET, we also provide information extracted from visuals, which is one of the core components of diplomatic action and public diplomacy.

We also contribute to the existing literature by placing a growing focus on computational image analysis.^41–44^. Politicians and diplomats strategically use this visual online content to enhance their narrative in the public space^11,45^. From leaders or higher officials’ stances, objects (symbols and flags), human composition (gender, and number of people) are some important aspects of visual content. For example, a previous research found that China’s public diplomacy (through visuals) has a positive effect on shaping the opinion of citizens^46^. Within the scope of GlobalDiplomacyNET, we particularly focus on the gender dimension of diplomatic news, which has been a significant topic in recent years^47,48^. Despite the diverse spectrum of gender, we follow the literature and define gender as man and woman^49^. We adopt this binary framework for empirical parsimony to address recent debates.

While we have data spanning different time periods for various countries, the time series data for certain nations (such as Russia, the United States, and Japan) prove to be particularly valuable for annual dyadic aggregation. For instance, we collected diplomatic news of these countries going back to the 2000s as shown in Fig. 2, extracting more information that social media data cannot offer. This is the point where researchers can integrate our data with existing dyadic datasets for different domain-specific purposes. Examples of such inferential uses can include political similarity (e.g. UNGA Voting Similarity^50^, trade partnership, under-reported global finance datasets^41^, leader visits^34,51^, interstate disputes ^52^, governance indicators^53–55^, global surveys (e.g., Gallup World Poll), conflict intensity^56,57^. However, we can point research directions where social media is an important medium for sharing diplomatic news and leaders’ views, leading a term *Twiplomacy*. In addition to official websites, the current literature also suggests leaders’ social media presence is effective and visible for diplomacy^58–60^. Censorship and shadow banning also leads politicians to construct their own online spaces and deplatforming of their followers like Trump’s Truth Social platform. These fringe communities can serve close followers, but their diplomacy related messages can have a different tone and framing. Zhang *et al*. compare partisan asymmetry for Trump’s activity on Twitter and Truth Social^61^. Cross-platform analysis between social media and official channels can offer interesting insights and our dataset can be beneficial to conduct such analysis.

## Methods

**Fig. 1:**
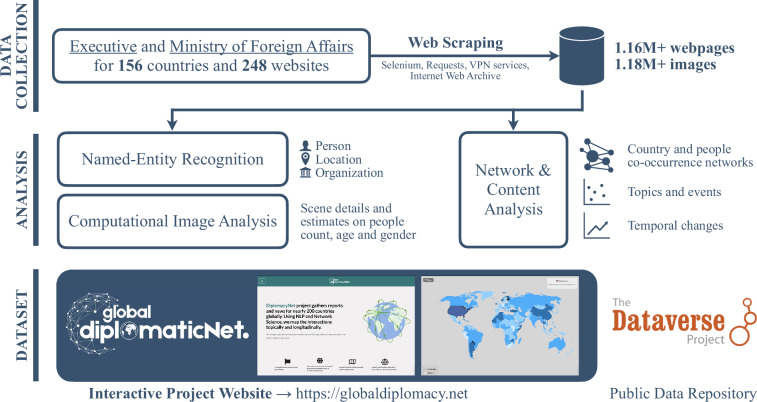
Schematic for the project modules. Project consist of three modules: i) data collection, ii) analysis, and iii) dataset and dissemination. We extracted content and images from 248 diplomatic websites. These dataset analyzed by using NLP and computer vision tools to conduct network and content analysis.

GlobalDiplomacyNETproject consists of several modules for data collection and analysis. In this section, we present the data collection methodologies and the approaches used to extract information from the gathered content. In Fig. 1, we present the schematic of the project to highlight the key components and their interactions with each other.

### Curation of country list and institutions

GlobalDiplomacyNET relies on the traditional (state-centric) definition of diplomacy, namely the states’ political activity conducted by official authorities who oversee and implement foreign policy^62,63^. GlobalDiplomacyNET’s country sample is composed of UN Member sovereign states, since a country’s diplomatic salience is affected by its recognition status. In other words, we excluded de-facto states such as South Ossetia and the Turkish Republic of Northern Cyprus (TRNC). However, we included the West Bank and Gaza Strip (mofa.pna.ps) and Taiwan (en.mofa.gov.tw) due to their high salience in the current geopolitical agenda. Particularly, recognition of Palestine as a sovereign entity have been one of the acute topics in international politics. We also excluded the British Overseas Territories due to their limited sovereignty.

**Fig. 2:**
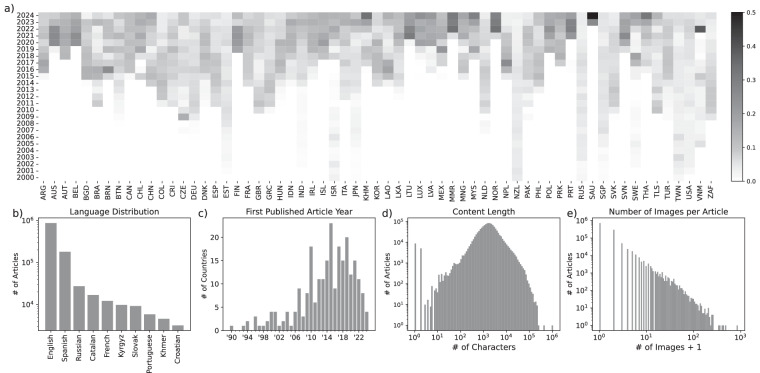
Dataset statistics. Collection of over 1.15 million articles summarized in terms of their temporal spans and content. We report temporal coverage for exemplar countries (**a**) and the first published articles for all countries collected for the project (**c**). Most of these reports written in English, followed by Spanish and Russian (**b**). Reported news can have just a title or as long as 10,000 characters (**d**) and they can contain images to support them (**e**).

Foreign policy implementation and diplomatic professions are primarily handled by countries’ ministries of foreign affairs under the supervision of the heads of government (chief executives). This definition also defines the principal actors of diplomacy as presidents, prime ministers, and foreign ministers^64^. We curated and compiled the GlobalDiplomacyNET dataset from countries’ ministry of foreign affairs and chief executive webpages. Considering the total of 156 countries and their 248 distinct websites, we encountered some exceptions in terms of their organizations. In case MoFA news focuses solely on foreign ministry activities, we collected data from both MoFA and Chief Executive websites. Some countries such as Liberia (mofa.gov.lr) and Maldives (foreign.gov.mv) had gaps in their news coverage. Observed temporal gaps can occur due to leadership changes, civil conflict and some external shocks such as COVID-19. We use Wayback Machine to fill those gaps when there is a *systematic* deletion of news. As a limitation, we were unable to find some conflict-affected and small states’ official websites during the phase of scraping process. In addition, some webpages did not respond during the scraping phase. In total, 40 UN Members are excluded in the current version of our dataset. In some other examples, governments’ official websites announce all executive and MoFA activities (e.g., Ireland, https://www.gov.ie/), filtering particular institutions or agencies. For semi-presidential systems, we scraped predominantly the president as the primary diplomatic actor. Since premier-presidential and president-parliamentary systems (e.g. Russia, http://en.kremlin.ru/) put a level of complexity, we only focused on president in such systems for conceptual and empirical parsimony. Prime-ministers in semi-presidential systems generally have a limited diplomatic salience when compared to president.

The number of websites exceeds the number of unique countries because we collected data from both MoFA and executive offices. For some countries, such as the United States, each presidential administration maintains separate archived webpages for both the chief executive and the secretary of state. During the research phase, we strictly complied with legal provisions within the scope of open-science practices^65^. In other words, GlobalDiplomacyNET does not include any personal data, and all data come from publicly available sources^66^.

### Collecting information from diplomatic websites by web scraping

Curation of the country list and the corresponding diplomatic websites present distinct challenges in collecting news content. These challenges emerge due to the technologies used in web development, completeness of historical records, or the anti-scraping methods employed to reduce automated traffic to the websites.

For this project, we have to develop distinct web scrapers for each domain, since all websites have a different structure. First, we identified the pages where the diplomatic news is listed either by some filters such as time and topic. Our scraping scripts collect URLs for news articles by visiting different pages or dynamically loading the next batch of articles by triggering JavaScript events. For static webpages, the use of standard web scraping tools like Python’s requests package was sufficient to iterate over the pages or send requests to API endpoints of the websites. Some dynamic websites require user interactions by clicking certain buttons or scrolling down the pages. In such scenarios, we implement emulators using Selenium library to mimic user behaviors. Once the URLs were collected, we visited and collected content from the websites as our second step.

Since some websites contain thousands of articles, platforms implement measures to prevent scraping such as CAPTCHAs to test authentic behaviors and protect sites from high traffic by using reverse proxy services like CloudFlare. On the other hand, we develop our custom scrapers to respond to such measures and send our requests from different IP addresses by using commercial VPN services. We noticed some government webpages restrict access or may not respond from particular countries’ IP addresses. Another challenge we face is the removal of content or websites altogether. When countries make changes to implement extra layers of protection or change their design, their content may become unavailable and custom scrapers also need reimplementation. For instance, Nepal (mofa.gov.np) updated their webpage in 2024, two years after our first scraping in 2022.

As we collect raw HTML content, we also extracted information from img tags to identify the pictures used in the articles and downloaded them for image analysis. Since websites use the same img tag for logos, icons, etc. we collected distinct paths for images for each website and repeating entries were assumed as design components and excluded. The remaining unique images downloaded and stored for analysis. However, there are also some countries like the United Arab Emirates which provide images loaded with Javascript in the website. In such cases, we were not able to collect and estimate image information from those websites without manual intervention, so we excluded them from the sample for images.

### Temporal analysis of diplomatic news

Each government demonstrates varying levels of transparency in disclosing diplomatic information. We have illustrated different characteristics of our textual data in Fig. 2(a), which demonstrates the availability of news articles and their concentration over the years. In the figure, we present data from 65 countries that the Lowy Institute includes in its Global Diplomacy Index, except for the European Union ^67^. This information is useful for several reasons. First, it signifies how countries structure and systematize their reporting activities based on their current webpage content. Figure 2(a) shows that several countries -such as Russia, the United States, Japan, South Africa, and the United Kingdom- have a more consistent pattern of information sharing. In cases where data coverage is limited, the Internet Archive can be used to complement information. Accordingly, we collected news from the Internet Archive for countries including Iran, the Netherlands, Denmark, and China.

Each government webpage has a specific language option. In Fig. 2(b), Spanish, Catalan, Russian, and French -as the main webpage language- are the most prevalent non-English languages in the GlobalDiplomacyNET. Notably, Russian news does not originate from the Russian Federation itself but rather from former Soviet republics such as Kyrgyzstan and partly Azerbaijan. Also, some countries in South and Central America show a strong tendency to publish Spanish news, which indicates that colonial ties can shape diplomatic communication regarding diplomatic news. This shows the impact of cultural proximity among these countries, which is particularly interesting for diplomatic communication. Figure 2(c) illustrates the initial year of publication of diplomatic communications (news). This reveals both the strengths and temporal limitations inherent in our dataset. A significant portion of news articles began in 2014, which still effectively covers the past 11 years. Figure 2(d) demonstrates the verbosity in the dataset, measured by the number of characters in each observation. Some countries, such as Cuba and Colombia, are particularly verbose compared to others. Countries with higher textual verbosity provide more input for the study of inter-state relations. This verbosity is significant also for images to gather more information from the content. Figure 2(e) provides information on visual communication strategies in international politics. The steep power-law distribution reveals that while most diplomatic communications use minimal visual content, a small subset incorporates numerous images.

### Detecting entities from news content

Named-entity recognition (NER) procedures are essential for text analysis, since they enable us to measure countries’ attention to specific regions, multilateral organizations, and persons. For our systematic analysis, we evaluated diverse family of models ranging from machine learning models trained specifically for this task or general use large language models (LLM) with few-shot prompting for NER task. We focused on three entity types: PERSON, COUNTRY, and ORGANIZATION.

Although most of the news was written in English, some news is collected in other languages. For language detection, we concatenated the title and article body and used the FastText model developed by Facebook AI Research, which supports the identification of 176 different languages^68,69^. This model also provides confidence level for detected languages and we only take into account when confidence level is above 0.5. When the detected language for the text is not English, we used Google’s Translate using googletrans package^70^. News that are longer than 2,000 characters split into smaller chunks and translated texts concatenated later. We preferred Google’s Translation API, since we also noticed some countries already integrated their JavaScript widget for adding different languages to their websites.

We evaluated the performance of several NLP libraries and models including NuExtract, Spacy and Gliner and LLMs with different parameter sizes including DeepSeek-R1, Llama, and Mistral. We used Ollama to facilitate local execution of these LLMs. These are among the most advanced LLMs and NLP libraries that demonstrate high performance for NER procedures^71^. Nine researchers from GlobalDiplomacyNET annotated 200 distinct diplomatic news items before the main execution process. These annotations were randomly assigned for evaluator assessment and later used to evaluate model performance. Additionally, each researcher annotated 20 extra news articles with 378 unique entities that were shared among all annotators to ensure consistency in the annotation process. Since annotators can detect entities by providing range of tokens, in some cases additional letters or words can be selected some annotators while others exclude those. Fuzzy string matching used to consider those entities as equivalent. Evaluation of inter-annotator agreement for span prediction tasks like NER is a complex task that needs to address challenges like chance agreement and class imbalance due to un-annotated tokens. In literature different measures like Fleiss’ kappa, Krippendorff’s alpha, and F1 scores for pairwise comparisons were proposed^72–75^. We use different measures to assess inter-annotator agreement. A straightforward analysis for Fleiss’ kappa offers score of 0.77, which demonstrates a substantial level of inter-annotator agreement particularly for political texts^72^. We also compute Krippendorff’s alpha in two settings since this measure can also take into account missing annotations: i) defining a separate label to penalize coder for missing an entity and ii) considering missing data for a coder if they are unable to detect an entity and not offering any label. Both cases lead to substantial agreement: 0.98 for recommended usage where we treat non-labeled tokens as missing annotation and 0.69 for more conservative estimate of treating missing entities as different labels. Analysis by considering the annotations of one annotator as the ground-truth and annotations of another as the predictions is used to calculate F1 scores^73,74^. For pairwise evaluation of annotations, we obtained F1 score of 0.78 ± 0.057. By incorporating different measures of agreement, we can suggest that manual annotations created to evaluate different NER tools are in a reasonable quality.

**Fig. 3:**
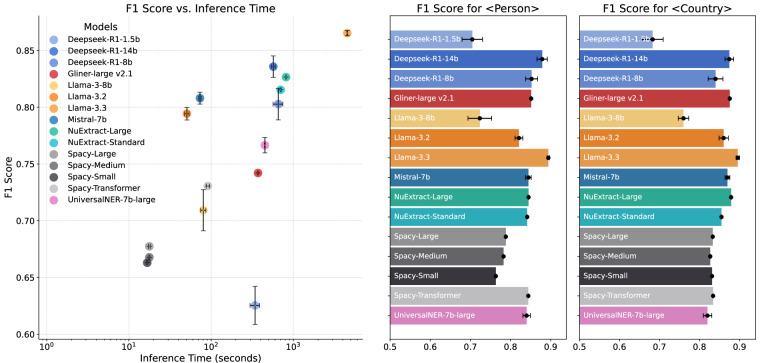
Model comparison for named-entity detection. Models were compared in terms of their inference time for 200 random news and detection performance with 10 repetitions to calculate error bars (**a**). Detection performance for PERSON and COUNTRY reported with models’ corresponding F1 scores (**b,c**).

The NER tool that is closest to our annotation is analyzed in Fig. 3. To quantify detection performances, we used F1 score measure. Since these models used to extract entities over a million documents, models execution time is also relevant. Llama-3.3 offers the best performance, but its inference time is almost an order of magnitude higher than other models. Because of this limitation, we selected DeepSeek-R1-14b^76^ for its efficient inference time and performance of 0.836 F1 score. These findings also corroborate LLMs performances measured by other scholars in different research areas^77^.

Since detected entities across documents can vary (e.g. Recep Tayyip Erdoğan, Tayyip Erdoğan, and President Erdoğan are all the same person), we use Wikimedia projects to resolve disambiguities and linking with a unique Wikidata QID (Wikidata unique identifier) that we will refer to as Wikidata QID. For each unique person entity, we collected responses from Wikipedia Search API^78^ and correspondingly Wikidata Search API and built a bi-partite network of entity strings and resolved Wikidata QIDs. We calculate the mapping between person entity strings and unique Wikipedia pages based on fuzzy-string matching between entity and Wikipedia page title as well as frequency of referral to Wikipedia pages. We later convert the Wikipedia URIs obtained from fuzzy matching to Wikidata QIDs. This way we assigned a Wikidata QID to all person entity strings that appear at least 10 times in our corpus. As for the countries and organizations we used Wikidata Search API directly for matching the entity string to page title. We used Wikidata’s structured knowledge base to compile the set of international organizations we wanted to include. After both country and organization entities were resolved, we validated the results manually as the entity space was quite small.

### Image Analysis

Advances in deep learning make systematic analysis possible to study large-volume of image data. To extract information from the images, we consider task-specific models for detecting people in the images and inferring their human characteristics such as gender. The Vision Language Models also offer opportunities to interact with the content through prompting with natural language. These models are computationally more expensive in terms of time and computational resources, but they are quite capable of answering simple image understanding questions.

The analysis focused on extracting information on gender and number of people shared on diplomatic news websites. The images were downloaded using the source URLs identified for unique images within the HTML files. For processing, we use pretrained models to extract information like YOLOv8 large model^79^ for detecting humans in the images. These human images were later analyzed by a pre-trained gender classification model on HuggingFace ^80^.

The exploratory analysis investigated two primary visual characteristics: overall gender composition and the number of people included in an image. This enabled us to measure and identify trends and disparities among countries in the portrayal of gender on diplomatic websites. In GlobalDiplomacyNETdataset, we provide statistics about the images and link those images with the articles shared in the dataset. We hope that the future research will be conducted with vision models and information extracted for the GlobalDiplomacyNETdataset. The potential to combine multiple datasets and investigate visual data is a promising research direction.

## Data Records

**Fig. 4:**
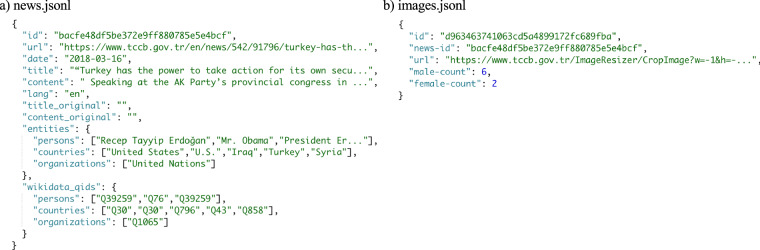
Samples from GlobalDiplomacyNET data records. Key-value pairs from the JSON files that contain records for news (**a**) and images (**b**).

Dataset offered by the GlobalDiplomacyNET project is available on Harvard Dataverse^81^. Aggregated data from 156 countries and 248 websites include 1,163,905 news documents and 1,187,152 images. In our dataset, we followed a specific naming convention to define folders and all relevant data places in that directory. Folder names composed of three components: <COUNTRY-CODE>_<TYPE>_<COUNT>. Country names are converted to standard alpha-3 country codes. We also distinguish websites for Executives (exec) and Ministries of Foreign Affairs (mofa) by <TYPE>. Since some countries may have separate websites for different presidents or time intervals, we also added <COUNT> key, which is only available when there are more than one version for that website. This way, users of our dataset can analyze particular countries of interest separately. In Fig. 4, we present samples records from the GlobalDiplomacyNET dataset. Within each country folder, we provide the following files:

news.jsonl: Each line in this file contains a separate news as JSON object. These objects contain a unique identifier (id), news details (url, date, title, and content). Since some countries publish articles in languages other than English, we add a key for the detected language (lang) and original content accessible with content-original and translated content is placed in the content. We also share detected entities (entities) and their corresponding unique Wikidata QIDs (wikidata-qid).

images.jsonl: For computational image analysis, we provide records that contains a unique identifier for an image (id), ID from news.jsonl to link images and news (news-id), and source of the image (url) as main information. We also provide results of the image analysis, detected number of males (male-count) and females (female-count) in the picture.

On the same directory as the country folders we also provide a summarizing file:

summary_statistics.xlsx: The summary statistics table lists all URLs associated with each website -as our sources- in the Harvard Dataverse dataset. We report the name of the country, its 3-letter ISO Alpha code, type of the website ("exec” or “mofa”), number of news, time-span of the news, fraction of non-English content, number of images, and female ratio.

## Technical Validation

### Validating named-entities within the news content temporally

Diplomatic texts mention different countries, regions, international organizations, politicians, and diplomats. Systematically identifying these entities is useful to capture governments’ geographical, individual, and multilateral attention. For instance, we can study co-occurrences of these countries or individuals to quantify the relations between these entities and it is also possible to filter these networks with temporal slides to investigate changes over time. This data can also test which multilateral institutions are becoming more salient over time. For instance, countries’ shifting focus toward or away from BRICS serves as an indicator of their attention to such organizations, showing possible geoeconomic adjustments. This is particularly timely and relevant given the growing regionalization in international politics^82^.

**Fig. 5:**
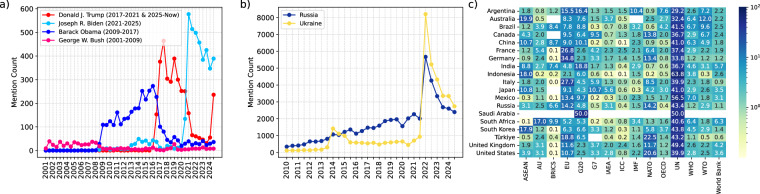
Global attention for named-entities over time. Entities extracted for PERSON (**a**), COUNTRY (**b**), and ORGANIZATION (**c**). We demonstrate number of mentions for the last four US presidents (**a**), Ukraine and Russia (**b**), and occurrences of Major International Organizations in the news of G20 Countries (**c**).

Figure 5 illustrates how often various named entities appear in our collection for interesting exemplary cases. Figure 5(a) tracks mention frequencies for the last four U.S. Presidents -Joseph R. Biden, Donald J.Trump, George W. Bush, and Barack Obama- revealing that each has distinct spikes in coverage corresponding to their presidential terms. It is significant that countries (other than the US) mentioned Obama even after his presidential tenure, which is not the case for George W.Bush. Donald Trump’s first presidential term attracted a sharp surge in diplomatic attention. However, after his term ended, this attention decreased sharply. Fig. 5(b) compares Russia (in blue) and Ukraine (in yellow) mentions, showing significant increases at key geopolitical moments (e.g., Ukraine’s sharp rise around 2014-2015 and again in 2022). Diplomatic news often contains early signals that precede actual formal conflicts. For instance, a surge in the monthly or daily mentions of Ukraine ahead of 2022 might reflect an escalation in rhetorical salience long before an actual invasion is triggered. Figure 5(b) also shows that mentions of Ukraine and Russia by other countries started to increase before the invasion. Researchers can adjust country-attention according to their own research in different regional contexts such as Taiwan, West Bank and Gaza, and others.

Finally, Fig. 5(c) provides a heatmap of major international organizations (columns) as referenced by different G20 countries (rows), where cell colors indicate the intensity of mentions (ranging from lower mentions in lighter shades to higher mentions in darker shades). The United Nations (UN), NATO, the European Union (EU), and The Association of Southeast Asian Nations(ASEAN) emerge as the most salient international organizations in the diplomatic communications of G20 countries. A high frequency of the UN mentions are expected, and Mexico, United Kingdom, Indonesia, South Korea emerge as the countries that mention the UN most. A prominent example is Russia, Turkiye, United States and Canada, and Germany, which mention NATO at a higher rate than other countries. Indonesia and India are the countries that refer to NATO the least in their diplomatic communications. The ASEAN is also mostly mentioned by Asian countries. Moreover, as a non-ASEAN partner country, China’s diplomatic communications demonstrate remarkable focus on this regional organization. In China’s diplomatic news coverage, ASEAN appears alongside other significant international bodies such as BRICS, G20, the United Nations, and the European Union, which can show China’s strategic prioritization of Southeast Asian regional engagement. It is also significant to mention that the WTO is mostly referenced by Australia, Argentina, Brazil and Mexico, which might demonstrate middle powers’ export oriented concerns. These analysis offer meaningful interpretation of temporal patterns of named-entities identified within diplomatic texts for domain experts. Information extracted from GlobalDiplomacyNET can be used for future research, and researchers can also utilize full text provided with the dataset for more in-depth analysis.

### Computational Image Analysis

In addition to textual verbosity, we also analyze governments’ practices of sharing images from diplomatic events and press releases. Researchers can pose several questions to study the information conveyed through image content. To minimize potential bias arising from insufficient data in this validation study, we excluded countries with fewer than 100 images. This threshold was set based on the distribution of images available per country, which ensures the statistical reliability and representativeness of the results. It is important to underline that we only collect images if the news HTML contains an <img> tag. Images embedded in JavaScript are therefore not captured.

**Fig. 6:**
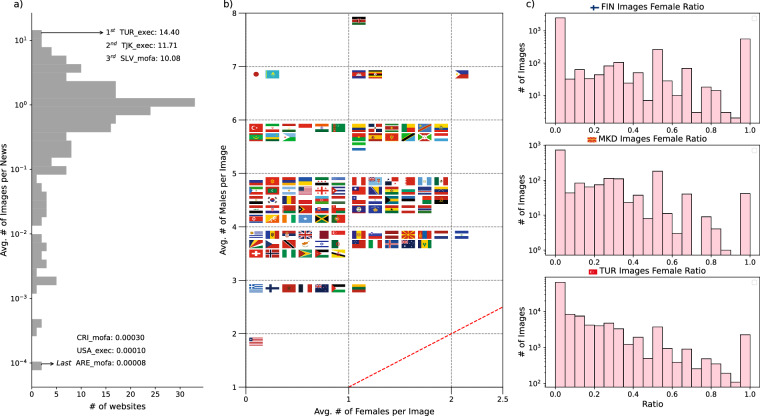
Analysis of images and participation in diplomatic events based on gender. 1.18 million images summarized in terms of their frequency per news (**a**). Average number of males and females across countries (**b**). Selected countries’ image distribution and gender fraction (**c**).

Based on Fig. 6(a), we find that countries such as Türkiye, Tajikistan, and El Salvador have the highest number of images per news. These statistics are an aggregation that originates from both the ministry of foreign affairs and chief executive websites. We generally detected that chief executive webpages tend to have a higher number of images per news article when compared to the ministries of foreign affairs. We also want to point out that some of these countries, such as Türkiye, chief executive webpages publish information on the domestic presidential agenda. In such cases, diplomatic news is blended with domestic issues under certain circumstances. Indeed, some of these pictures come from public meetings and government propaganda, which can substantially inflate the number of images. Therefore, such circumstances do not directly imply the “diplomatic” side of image sharing. However, this news content is in English and directly communicates with the global audience. We can interpret this finding as part of public diplomacy in which countries signal their political resilience and public support. Moreover, if researchers desire to acquire visuals (or texts) related to just foreign politics from chief executive webpages, they can use GlobalDiplomacyNET’s country-entities to filter foreign-related visuals and news content.

Figure 6(b) shows a significant trend in gender-dimension of image-sharing. The plot shows countries relative to the diagonal line, which represents absolute gender-equality.. The figure shows that most flags lie above the diagonal, which indicates that in the vast majority of these news articles, men appear more often than women. Countries are concentrated between 4 and 5 men per image. We can suggest that the presence of women in diplomatic and political visuals is always less than men’s in all countries in our dataset. El Salvador and Lithuania are the closest countries to the equality line. European and Latin American countries seem to include more women in their diplomatic news as seen in Fig. 6(b). Researchers can employ our image analysis across multiple time periods and geographical contexts by appealing different variables. For instance, the democracy and gender-dimension of diplomacy have been significant issues in the literature, where GlobalDiplomacyNET image analysis can leverage such domain-specific research fields. Our current study’s scope is limited to 2024 data, but future work could reveal valuable longitudinal trends and cross-cultural patterns on countries’ image-sharing habits. Figure 6(c) reveals the distribution of the female ratio for three examples including Finland, North Macedonia and Turkey on a log y-scale. In all three countries, images with many men are far more common than images with many women. Finland is relatively more balanced but still male-skewed. Türkiye has the longest right tail for men, because the crowd events reaching more than 30 men per image on average. Moreover, women presentation generally decreases if images include a crowded population.

### Validating network of people from entity co-occurrences

Network science offers tools to analyze interactions between different entities, here we investigate co-occurrences of people with the same news. This network representation points clusters of individuals that reflect mostly the geographical associations, but also their interactions due to diplomatic agenda and shared organizational memberships. One can also study such network overtime to investigate when certain world leaders or foreign ministers begin to appear together in diplomatic communications. Such analysis may signal the formation or strengthening of bilateral ties. Conversely, an interruption in mentions of a particular individual following a policy dispute can forecast increased tensions.

**Fig. 7:**
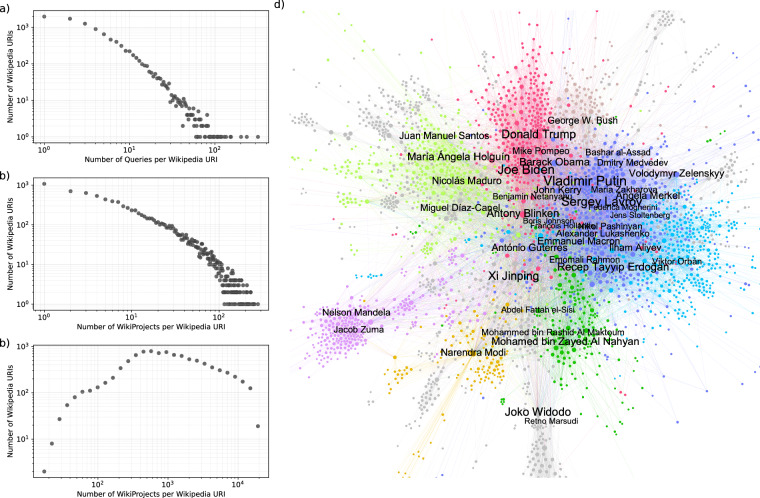
Network analysis of individuals through Wikipedia URIs. Same Wikipedia IDs can be referred with different ways in the news and their distribution follows a power-law (**a**). To assess the popularity of each person, we collected Wikimedia project for different languages (**b**) and also calculate the content length distribution for English page (**c**). The co-occurrence network presents people in the nodes and their interaction frequency represented as edge weight (**d**).

In Fig. 7, we present results on co-occurrence network of individual people appeared on diplomatic news. Nodes in this network correspond to different Wikipedia URIs and links between them capture the frequency of appearing on the same news. Use of the Wikipedia API helped us disambiguate different ways to express same political figures in the text and map them into single entities. We plot the distribution for the number of unique entities mapped to particular Wikipedia URI in Fig. 7(a). Most political figures mentioned in a few different ways; however, some individuals were mentioned more than 100 times. When we inspect those individuals, we noticed they take multiple titles over the year or they adopt using multiple family names to show their ancestry. For instance, President of the United Arab Emirates Mohamed bin Zayed Al Nahyan is most frequently referred to as “his highness sheikh mohamed bin zayed al nahyan”, “h.h. sheikh hamed bin zayed al nahyan” or “sheikh mohammed bin zayed al nahyan”.

It is also known that not all political figures are equally represented. Some of them are more prominent than others, and we can quantify that by analyzing how many different Wikimedia projects they have a page (see Fig. 7(b)) and what the length of the content is in the English edition of Wikipedia as shown in Fig. 7(c). When we inspect the top leaders based on the different Wikimedia project they covered, US presidents like Donald Trump, Barack Obama, and Joe Biden were among top-10. We also detected founding leaders like Nelson Mandela and Mustafa Kemal Atatürk in the list. One can collect further information about those figures and only analyze the politically active individuals.

In Fig. 7(d), we visualize the largest connected component of the co-occurrence network. We highlight the nodes corresponding to politicians who are among the top 30 for either having the highest degree centrality or node strength. This network encompasses all years in our dataset and provides insights into international political trends. Clusters correspond in part to regional groupings. For instance, Latin America’s political figures are concentrated in the green nodes, such as Juan Manuel Santos, Angela Holguin, Nicolas Maduro, and Miguel Diaz-Canel. This co-occurrence can result from these leaders’ meetings, political interactions and mentions in the news. However, diplomatic news includes cables and many other forms of person-mentions for political figures. António Guterres emerges as a central political figure in the network, which makes sense due to his position as the General-Secretary of the United Nations.

A co-occurrence network can also be related to political figures’ salience instead of their regional position. Zelenskyy, Medvedev, Bashar al-Assad, Putin, Angela Merkel, and Lavrov mostly co-occur together in countries’ foreign news. In this regard, Al-Assad -Syria’s overthrown leader- does not emerge as part of the Middle-Eastern cluster in the network. In other words, the mentioned characters’ deviation from their regional cluster seems to parallel their corresponding salience in global affairs. Within the United States’ high-level representatives, Anthony Blinken is closest to the epicenter of the co-occurrence network, meaning that his name co-occurs with other countries’ political figures more than other American politicians. It is also interesting that China’s president, Xi Jinping, is very close to the center of the network, implying China’s global diplomatic hinterland. The network analysis offers tools for validating how different entities interacts and richness of the data offered in GlobalDiplomacyNET. Our dataset can be used to study temporal dynamics between political figures or in combination with other data sources about political leaders and regimes to investigate novel research questions.

## Usage Notes

We also developed an interactive website for our users to inspect different dimensions of our analysis and access our dataset. Our GlobalDiplomacyNET website is online at globaldiplomacy.net. We plan to keep the dataset updated by executing the data collection pipelines yearly. Since websites can change their design, we plan to adjust those changes in our code base. One of the known limitations of diplomatic websites is their limited capacity to archive their content. Most websites delete older posts for no transparent reason or miss them in website updates. Wayback Machine of the Internet Web Archive could be an alternative; however, most of the sites only have snapshots of their landing pages and links to diplomatic news were not captured by the crawlers.

## Data Availability

We released the entire GlobalDiplomacyNET dataset on Harvard Dataverse (10.7910/DVN/HYJDE0). Users can download the full text and additional content that we offer from diplomatic news such as entities, image features for different countries websites. All data are public records and were scraped from publicly accessible webpages of ministries of foreign affairs and chief executive offices, whose URLs are listed in the Summary Statistics table. All source webpages are publicly available without access restriction. No personal data were collected, and our usage and data mining practices strictly comply with the open science principles, re-use provisions under EU Directives 2019/1024^66^, 2019/790^83^, and other recommendations from OECD Member Countries, including the UK, and commonwealth countries (Canada, Australia).

## Code availability

We provide a GitHub repository to share codes for the analysis conducted in this paper: github.com/ViralLab/GlobalDiplomacyNet-Dataset. For webscraping we developed custom codes for each website. Selenium and requests libraries were used to retrieve data and BeautifulSoup4 package used for parsing HTML content.
